# Eighth Annual Meeting of the Mississippi Valley Association of Dental Surgeons

**Published:** 1852-10

**Authors:** 


### ARTICLE VIII.

### *Eighth Annual Meeting of the Mississippi Valley Association of Dental Surgeons.*

This society met according to adjournment, at the Ohio College of Dental Surgery, at 10, A. M., Wednesday, September 8, 1852.

In the absence of the other presidents, Dr. W. H. Goddard, third vice, took the chair, and called the meeting to order.

The following gentlemen were present and answered to their names: Drs. Goddard, McCullom, Baxter, Hunt and Bonsal.

On motion, Dr. Bonsal was appointed Secretary pro tem.

Neither of the members of the executive committee being present, on motion Drs. Hunt and Baxter were appointed a committee to prepare business for the meeting. On motion, adjourned to meet at 10 o'clock to-morrow.

*Second Day.*—Society met at 10, A. M. Drs. Ulrey, first vice, in the chair. Present—Drs. Goddard, James Taylor, Ulrey, Van Emon, McCullom, Taft, Bonsal, Baxter, Hunt, Leslie.

Drs. Watt and Hammill being present, they were invited to participate in the transactions of the society.

Minutes of yesterday were read and approved.

The committee appointed to prepare business being called upon, reported as follows:

#### REPORT.

*To the Miss. Valley Association of Dental Surgeons:*

The committee appointed to prepare and arrange business for the action of the society, would report that they recommend proceedings as follows:

1st.—Report of examining committee.

2nd.—Reports of officers of the asssociation.

3d.—Reports of standing and special committees.

4th.—Addresses before the association by gentlemen appointed for that purpose at the last annual meeting.

5th.—Consideration of any subjects which may be deemed of peculiar importance, in relation to operative or mechanical dentistry.

6th.—Your committee would also respectfully submit to the association, that some well defined action on the subject of dental patents be taken by the association, as your committee believe, that the profession abroad and the public labor under mistaken impressions of the views of the members of this association on that subject, and your committee would offer for adoption the following resolution:

*Resolved,* That a committee of three be appointed to present such resolutions on the subject of dental patents, as they may think advisable, and that their report be made the order of the day for 2 o'clock, P. M.

7th.—Your committee would also offer the following:

*Resolved,* That a committee of three be appointed to take into consideration the propriety of dissolving this association, and report at 12 o'clock.

A. M. Hunt,
J. W. Baxter, } *Committee.*

On motion, this report was adopted, and the chair ordered to fill the committees, which he did by appointing on patents, Drs. Taft, Leslie and Van Emon. On dissolution, Drs. Goddard, Hunt and James Taylor.

Dr. McCullom was called upon for his paper on the use of the file. The doctor remarked, that he had not prepared a paper on the subject; he thought the matter so well understood, there also was so little of it, and he had so little new on the matter, that he did not think it worthy of being written upon, but was ready to give his views extempore, which he proceeded to do briefly.

Dr. Van Emon was next called upon for the paper he was appointed to prepare for the previous meeting. The doctor begged to be excused; the matter had entirely escaped his memory until reminded of the fact a few days ago by Dr. Leslie. He, however, did not see that anything could be written on the blow-pipe. Members generally knew the use of it pretty well. It was true, he had made an improvement on the blow-pipe, but he could not see the consistency of detaining them with a discussion of what he had resolved to secure to himself. However, what he had to say, he could say without writing. The doctor then spoke of the form he thought best to give the point and the curve of an ordinary blow-pipe; he also spoke of the two well known powers of a blow-pipe jet, viz. oxydizing and reducing; he also had something to say of a right angle and an obtuse angle, in describing the curve of the pipe, but his idea here we could not get; perhaps if he had written, it might have been clearer.

The committee on dissolution being prepared to report, presented the following:

Your committee respectfully recommend the adoption of the following resolution:

*Resolved*, That when this society adjourn, it stand adjourned sine die.

On motion, report was accepted.

A motion being made to adopt it, discussion was opened upon it by Dr. Leslie. He contended, first, that its consideration, just at that time, was premature; for one, he wished to see what action would be taken on the subject of patents, before he could determine how he stood on this question. If the society would not come on the right ground on this subject, for one he would say, the sooner the association was dead and buried the better. Support the patent system and our annual conventions would be worse than useless; they would prove as they came round, each to be a farce, and as useless as the present has been, so far. We would have the same thing repeated we have heard from one member to day, viz. "that it would be useless to detail that to them which he expected to *secure* to himself."

I cannot but admire the candor of the gentleman in giving this as a reason for not filling his appointment. I would commend his *consistency* also to the attention of all dental *patentees and their abettors.*

Dr. Goddard sustained the resolution, on the ground that it was impossible to obtain the co-operation of the members of the profession in the Mississippi Valley. There were also a number of the members of the association who failed in attendance, and it seemed useless to continue to meet for no greater results.

Dr. Taylor thought that if we could not enlist more generally the professional gentlemen of the Valley, we had better disband. And this seemed to be the sentiment of Drs. McCullom, Hunt and Van Emon. The latter also wished to guard the society against what he considered an improper application, by Dr. Leslie, of one of the reasons given by himself for not presenting a paper on the blow-pipe.

On motion of A. M. Leslie, the further consideration of this resolution was laid over until five o'clock, to-day.

Dr. James Taylor presented the following report:

The editor of the Register would respectfully report, that he has attended to the duties of editing the Journal in as prompt a manner as he was able. That he has

| | |
|---|---|
| Received on subscription, . . . . . | $223 00 |
| " for advertising, . . . . . | 12 00 |
| | 235 00 |
| Deficiency, . . . . . . . . | 10 00 |
| Paid for publishing, . . . . . . | $245 00 |

Report accepted, and on motion of Dr. James Taylor, Drs. Hunt and Taft were appointed an auditing committee, to report this afternoon.

The examining committee reported the names of Drs. Watt and Hammill as applicants for membership, and recommended them as worthy of fellowship. After balloting they were declared unanimously elected.

On motion of Dr. Bonsal, adjourned to meet at 2, P. M.

*Afternoon Session.*—Society met at 2, P. M. The special order being the report of the committee on patents, their report was presented, discussed at length, and finally amended so as to read,

The committee appointed to report on the subject of patenting improvements in dentistry, respectfully report,

That inasmuch as our profession is a department of a liberal profession, and inasmuch as the wisest, most talented and liberal men amongst us cherish most cordially the free interchange of ideas and modes of practice, your committee recommend the adoption of the following resolution:

*Resolved*, That it is now, and always has been the sentiment of this society, that it is *derogatory to the professional character* of any of its members to *patent any dental instrument or mode of practice.*

J. Taft,
A. M. Leslie, } *Majority of Committee.*

On this report a lengthened and somewhat lively discussion took place. The main feature of change made by the amendment in the report was, by striking out a portion of the resolution where it was declared that the procuring of a patent by any member would be sufficient cause for expulsion. This was stricken out on motion of James Taylor.

Dr. Taylor remarked, that he was not willing to go so far as the committee would seem to want. He perceived the resolution was the same as that presented a year ago. He was not ready to cut off a member who had procured a patent. He had also little to say on the subject of patents now, as it was well known he had recently, through the press, given his views at length on that subject; he still thought as he did then; he had seen no cause for change; he admitted his views, as expressed at the second annual meeting of this society, had undergone some change, but not to the extent some thought. He had then advocated the propriety of allowing members to patent a mechanical improvement, which could be sold wholesale and retail, and the public not be injured. He was now ready to go a little further than that; he thought now we might safely concede the right to patent any thing pertaining to mechanical dentistry. We might do this, and still the welfare of our patients be perfectly safe as regards the preservation of health. This was the point guarded against in the medical profession; this satisfied the members of that profession, and he thought it ought to satisfy us. It is also well known that the working of the patent laws was that the discoveries of the day were made public; it is not true that there is any thing secret about a patent.

Dr. Leslie was opposed to cutting off that portion of the resolution which declared the procuring of a patent sufficient cause for expulsion; he thought it high time to act on the matter; he freely granted that if this passed we would be bound to apply it, and for one he fully intended it should be applied whenever applicable; certainly we needed it. The resolution of the committee is identical in spirit, and nearly in words with the one presented by Dr. Edward Taylor, at the second annual meeting of this society, and which was so ably supported by several members, *especially* Dr. James Taylor. Let me read the Taylor resolution for you again. "That any member of the society who may patent any instrument or improved mode of practice, may be subject to expulsion from the society." Here let me notice and correct a confused statement that has appeared, (in a paper miscalled "a Review.") There the reader is led to believe, that as this resolution was, after discussion, laid on the table until next day, and next day withdrawn, that *no* action was had upon it. The same idea is also couched in the question just put by Dr. Taylor—"did this resolution pass?" I say no; but if the doctor will just look at the substitute offered in its place, he will see that it is the same resolution amended, so as to be more lengthy, but less useful, by having the expulsion clause stricken out, and *further* action deferred; that is, the question whether or not we should *expel* for this act derogatory to the professional character of any member, was deferred until next meeting. The folly of thus temporizing has been clearly shown in this case; had the society stuck by the original resolution, it may be, we would have saved a member from a second time doing that we had declared unprofessional.

I trust, therefore, the society will not amend as Dr. Taylor proposes. To adopt the balance only of the resolution, would be but to repeat what we have already said: let us adopt the whole and apply it. We have had theory on our records long enough, now give us the practice.

Dr. Van Emon could not approve of any part of the report, if it be true, then our legislators, who made all our patent laws, must have erred, but he could not think this; he believed that every man had a perfect right to secure to himself the result of his labors, it was folly to suppose a man would make a useful invention the property of the public, after he may have spent years, and much money, in the accomplishment of his design. He thought that should the sentiment of the report prevail generally, that we would have no discoveries announced, he thought that if we had no patent laws, we would have little or no improvements made, he viewed a sound system of patent laws as the great prompter to improvement, unless a man saw in advance that his inventions would be secured to him, he would never labor to effect them. He could not see why we should not recognize these general principles as applicable to dentists, he could not see how we could stop short of applying it to every improvement that man can make, he could not even see the impropriety of patenting a medicine; why should not the medical man be remunerated for his years, or months, of toil in producing an article, safe and infallible in the cure of any particular disease, as well as the surgeon who has made some new instrument, or the mechanic who has made some new machine, they were each deserving of a reward, and unless there was some such reward as the patent system, valuable secrets and inventions would die with the discoverer. It surely will not be said, the medical man is less liberal than the mechanic, he surely will not refuse to pay his fellow practitioner for the privilege of using that which is essential to the well being of his patients. He thought the operation of a patent, in securing the rights of an inventor, were the same as the operation of a copyright in securing the laborer in the literary field, from piracy. In answer to an inquiry, he said, he would have prepared a monthly report had he enjoyed time enough, but this he had not; he, however, would say that it would have been directly opposed, in sentiment, to that of the majority. The doctor also thought, that the patent was the very best means of preventing secrets in practice, for it was well known, that so soon as a patent was procured, the whole matter was made public.

Dr. Leslie thought, that Dr. Van Emon had now given his friend, Dr. Taylor, rather a hard task to accomplish. Dr. Van E. cannot see the nice line that separates the right to patent something mechanical in dentistry, from something chemical in dentistry, or something instrumental in surgery, from something medicinal in physic, and, I confess, I am as blind as the doctor, I cannot see the line of distinction claimed by Dr. Taylor to exist. I cannot see how any member of our so called liberal profession, may have claims to a reward for his labors in producing an improved set of teeth, and not also retain the right to a reward of labor in a discovery of a dentifrice, *because*, I view a perfectly constructed set of artificial teeth, of far greater importance to the well being of the wearer, indeed, I may say, the *preservation* and enjoyment of *life*, than any and all dentifrices which can be produced. I believe that it may be safely said, that the hygienic effects of mechanical dentistry (improperly so called, I think,) far transcend, when rightly executed, those of chemical dentistry; so that you see, I would not degrade even this part of dentistry, by calling it *merely mechanical*, and *thus* justify a patent in this branch, "now sir, we have always regarded this mechanical view as the great curse of the profession, it is this which retards the progress of dental science." I must, however, leave it to Dr. Taylor to explain to Dr. Van E. how it is that he may patent a blow-pipe, but, as a member of a liberal profession, he must not patent any discovery he may make in dental chemistry, or in medicine.

There is one thing further on the subject of dental patents I would call the attention of the society to, and that is, *there cannot now be pointed out one single improvement made on dentistry*

*that is now used successfully in practice, which has ever been legitimately protected by a patent.* Patents there have been, as the history of them, by Geo. W. Kendall, shows, but improvements they have not been, as experience fully settles; verily our patent laws have helped the science of dentistry on amazingly; just about as the monkey helped the cats to divide the cheese, that was helped himself. Still one thing we all must acknowledge, viz. the practice of dentistry has made valuable, and rapid inprovement in the last twenty, indeed, I may say ten years, and whence came the improvements? I claim *all the valuable and real advancement* made by the profession, has resulted from the labors of those men who have always viewed it as unprofessional to patent anything pertaining to the practice of dentistry, and who have ever cherished a liberal feeling in their intercourse with their fellow practitioners. If the opposite of what I have here stated can be shown, I call upon the possessor of such information, to produce it, I challenge the abettors of patents to the proof. A point which Drs. Taylor and Van Emon have endeavored to make, is, that patenting is to be justified on the ground that the inducement to hold secrets is removed. There being no secret under the patent laws, the procuring of a patent at once spreads a knowledge of the invention before the public. This idea has been harped upon before, and it is a little astonishing how a sensible man can make the statement once, to say nothing of repeating it. A glorious specimen of liberality truly. The patentee is *required* to file a description of his mode and means made use of, and because another clause secures the rights of any individual to a copy of the same, which, however, he *dare not use until the expiration of the period of the patent*, therefore, there are no secrets approved of by those approving of patents—sage conclusion truly. What generosity is there in telling a man how he may swim, while at the same time you hold the cord that binds his hands and feet. This is tantalizing with a vengeance. I know of no case parallel to it but an imaginary one by Dickens, personified under the character of Quilp, but the climax of his sham philanthropy is seen, I think, when he seats himself just outside a circle, the diameter of which is twice the length of a dog's chain, and prides himself in making a generous offer of his (animate) clay for dental experiment, bite! bite! he says, why dont you bite, "I make no secret of the structure of my body." I commend the study of Quilp to dental patentees and their abettors.

Dr. Hart remarked, that he thought that it was necessary that this society should, at the present time, take some decided action on the subject of patents, he thought the position of the association on the matter is misunderstood, he must say, he held a different view of the position of the medical profession, on the subject of patents, from what had been expressed by others. He had been connected with several medical societies, at one time five, now he is a member of three, in all of them, he was certain that the patenting of any thing pertaining in any way to the profession, whether instrumental or medicinal, would not be tolerated, and such, he believed, was the sentiment of the medical profession as a whole; certain, he was, that the American Association of medical men was opposed to it. On the subject of patenting in our profession, his mind was fully made up, he would go against patents on mechanical dentistry, as well as their application in medicine. The doctor did not seem to see that ill defined line of separation so much needed at the present time.

Dr. Taft also thought that the society should take such action as would manifest a disapproval of patents in dentistry. He had nothing to say against the patent laws, in general; they, no doubt, were very good for society, in general. He had nothing to say against a dentist patenting anything not essentially connected with the profession; but he certainly thought this society should clearly disapprove of all dental patents.

The question being taken on the amendment of Dr. Taylor, it was carried by a majority of one or two. The question then being on the adoption of the report and resolution, as amended, it was carried without a dissenting vote.

Dr. Taft being called upon for his paper on dentition, read an unfinished article on this head; and on motion, he was requested to continue it.

The auditing committee presented the following report:

Your committee have examined the books of the editor, and find the receipts, as they enumerate them, to be—

| | |
|---|---|
| Received on subscriptions, . . . . | $206 00 |
| For advertising, . . . . . . . | 12 00 |
| Deficiency, . . . . . . . . | 27 00 |
| Cost of publishing, . . . . . | $245 00 |

A. M. HUNT, J. TAFT, } *Committee.*

On motion, the report was accepted.

The following resolution was then presented:—

*Resolved,* That we tender to Dr. James Taylor, the back numbers of the *Dental Register,* together with the books received in exchange, and the subscriptions now due on the *Register,* as a remuneration for his labors, as editor, provided he pays the balance due the publisher, and that he be requested to continue the publication of the *Dental Register,* at his own expense.

Dr. Taylor having expressed his willingness to comply with the resolution, it was adopted.

Dr. Leslie reminded the chair, that there existed a standing committee, which, it was possible, was prepared to report. He therefore suggested it be called up.

Dr. Taylor remarked, that he believed he understood what Dr. Leslie referred to, viz. the committee on Dr. Drake's questions. He said, the committee was not yet fully prepared to make a report; a difficulty in the way, was the distances which separated the several members of the committee, and prevented consultation. This would soon, in part, be remedied, as Dr. Berry intended moving to Cincinnati soon. He had himself prepared the greater part of the report; and he thought he would soon be able to finish it, and place it in the hands of Drs. Goddard and Berry, for examination. It would certainly be ready in time for the publication Dr. Drake designed to use it in.

Whereupon, on motion, the committee were instructed to send their report, when finished, to Dr. Drake, for publication, and also to publish it in the *Dental Register.*

The regular order being the consideration of the resolution on dissolution, the chairman of the committee asked, and obtained leave to withdraw it.

On motion, adjourned to meet at Dr. Taylor's office, at half-past seven o'clock, P. M.

*Evening Session.*—On motion of Dr. Goddard it was *Resolved,* That this association invite its members to be present, at its next annual meeting, all discoveries and improvements which they may have recently made in dentistry; and this association will award a gold medal to him who shall produce and demonstrate the most valuable improvement and invention, the same to be decided by a majority of those present.

On motion of Dr. Goddard, those gentlemen present, not members of the association, were invited to take part in the considerations of the evening.

On motion of Dr. Baxter, it was *Resolved,* That this association will, in future, after the payment of its necessary expenses, devote the balance of its funds to the formation of a fund for the purpose of rewarding any valuable improvements appertaining to the profession of dentistry—to be decided at each annual meeting.

On motion of Dr. Taylor, it was *Resolved,* That this association award a gold medal, worth twenty dollars, on the best set of extracting instruments exhibited at the next annual meeting.

On motion of Dr. Goddard, the constitution was altered, so as to have but one Vice-President.

The society having resolved to go into election of officers, after a somewhat protracted balloting, the following gentlemen were selected:

President, J. Taft; Vice-President, A. M. Hunt; Recording Secretary, A. M. Leslie; Corresponding Secretary, Samuel Griffith; Treasurer, Chas. Bonsall; Examining Committee, Drs. Berry, Goddard, and McCullom; Executive Committee, Drs. Ulrey, Watt ,and Baxter.

Dr. Van Emon having, through the secretary, presented his resignation of membership, it was, on motion of Dr. Goddard,

*Resolved,* That his resignation be accepted so soon as he is clear of the books.

On motion of Dr. Bonsall, *Resolved,* That when we adjourn, we do so to meet in Cincinnati, on the day succeeding the annual commencement of the Ohio Dental College, and that hereafter our annual meetings shall be held at that time.

Drs. Watt, Hamill, and A. M. Hunt, were appointed to prepare papers to be read at the next meeting.

Dr. Leslie having been furnished, by Dr. Hunter, with copies of his recipes and specimens of his new style of work, laid them before the society; when, on motion of Dr. Hunt, it was

*Resolved,* That the thanks of this association be tendered to Drs. Hunter and Leslie, for the exhibit just made.

Dr. Hunt also remarked, that did not the lateness of the hour prevent it, he would propose something more.

On motion, the eighth annual meeting was now adjourned.

The members separated in good feelings, and with the conviction, generally expressed, that there is now a more useful future opened up for the society, and a better prospect existed for professional improvement, than formerly.

*Messrs. Editors:* I have sent you this report, in order that it may have a wide circulation, and that the profession may know, through the pages of the *Journal,* what we are saying and doing out here in the west. A. M. L.

